# Case report: The devil was hidden in the mastocytes – an unusually fulminant case of indolent systemic mastocytosis in a 45-year-old patient, missed for almost 20 years

**DOI:** 10.3389/fimmu.2023.1134587

**Published:** 2023-02-10

**Authors:** Aristeidis E. Boukouris, Ioannis Michelakis, Dionysios Metaxas, Gianna Karapati, George Kanellis, Athina Lioni, Vasiliki Tzavara

**Affiliations:** ^1^ 1st Department of Internal Medicine, Korgialenio-Benakio Red Cross General Hospital, Athens, Greece; ^2^ Hematopathology Department, Evangelismos Hospital, Athens, Greece

**Keywords:** case report, indolent systemic mastocytosis, fulminant presentation, coma, symptom mitigation

## Abstract

Indolent systemic mastocytosis (ISM) represents the most common form of SM, typically following a slow clinical course. While anaphylactic reactions may come up in the life course of an ISM patient, these are often moderate and do not pose a threat to patient’s health. Here, we present an undiagnosed case of ISM with recurrent severe anaphylactic episodes following consumption of food and emotional stress. One of these episodes led to anaphylactic shock, necessitating temporary mechanical ventilation and intensive care unit (ICU) support. Besides hypotension, a diffuse, itchy, red rash was the only notable clinical finding. Upon recovery, we found abnormally high baseline serum tryptase level as well as 10% bone marrow (BM) infiltration by multifocal, dense clusters of CD117^+^/mast cell tryptase^+^/CD25^+^ mast cells (MCs), consolidating the diagnosis of ISM. Prophylactic treatment with a histamine receptor antagonist was initiated, resulting in milder episodes thereafter. Diagnosis of ISM requires a high level of suspicion; its prompt recognition and treatment are important in preventing potentially life-threatening anaphylactic episodes.

## 1 Introduction

Systemic mastocytosis is a rare (estimated prevalence: 1/10,000) (1) hematologic condition, characterized by increased proliferation and uncontrolled accumulation of mast cells in various organs, including the skin, bone marrow, liver, spleen and GI tract. Common manifestations include anemia, abdominal pain, pruritus/flushing and anaphylactoid reactions as well as neurocognitive symptoms (e.g., depression, mood changes, lack of concentration). Hepatosplenomegaly, lymphadenopathy and signs of anemia are often noted on physical examination (2). Disease severity and course can vary greatly depending on the degree of mast cell infiltration and resulting organ dysfunction. While aggressive SM and mast cell leukemia (MCL) (collectively termed advanced SM) are characterized by rapid progression and poor survival rates, less severe cases of SM (indolent SM; ISM and smoldering SM; SSM) follow a much less impactful course over several years (median OS: 28.4 years according to large ISM patient databases) (3), often making it hard to discern from other diseases. Diagnosis (**Table 1**) and classification (4) are based on the combination of a number of different findings: a) clinical (B-findings, indicative of high MC burden and involvement of various organs (bone marrow, liver, spleen, lymph nodes) without causing dysfunction *vs.* C-findings, indicative of SM-induced organ dysfunction (cytopenias, hepatopathy, hypersplenism, malabsorption, osteolysis)), b) serological (measurement of serum tryptase) and c) histological (tissue biopsy). Here, we present a rare case of ISM with fulminant presentation in a 45-year-old male that remained undiagnosed for almost 20 years, eventually leading to a potentially life-threatening event (anaphylactic shock, coma and intubation).

**Table 1:** SM Diagnostic Criteria (2021 update).

Major SM Criterion
Multifocal dense infiltrates of MCs (≥15 MCs in aggregates) in BM biopsies and/or in sections of other extracutaneous organ(s)
Minor SM Criteria
a. ≥25% of all MCs are atypical cells (type I or type II) on BM smears or are spindle-shaped in MC infiltrates detected in sections of BM or other extracutaneous organs
b. KIT-activating *KIT* point mutation(s) at codon 816 or in other critical regions of *KIT* in BM or another extracutaneous organ
c. MCs in BM, blood, or another extracutaneous organ express one or more of: CD2 and/or CD25 and/or CD30
d. Baseline serum tryptase concentration >20 ng/ml (in the case of an unrelated myeloid neoplasm, item d does not count as an SM criterion. In the case of a known HaT, the tryptase level should be adjusted)
*Establishment of diagnosis: 1 major + 1 minor OR 3 minor criteria*

BM, Bone Marrow; HaT, Hereditary alpha-Tryptasemia; MC(s), Mast Cell(s); SM, Systemic Mastocytosis.

## 2 Case presentation

**Figure 1:**
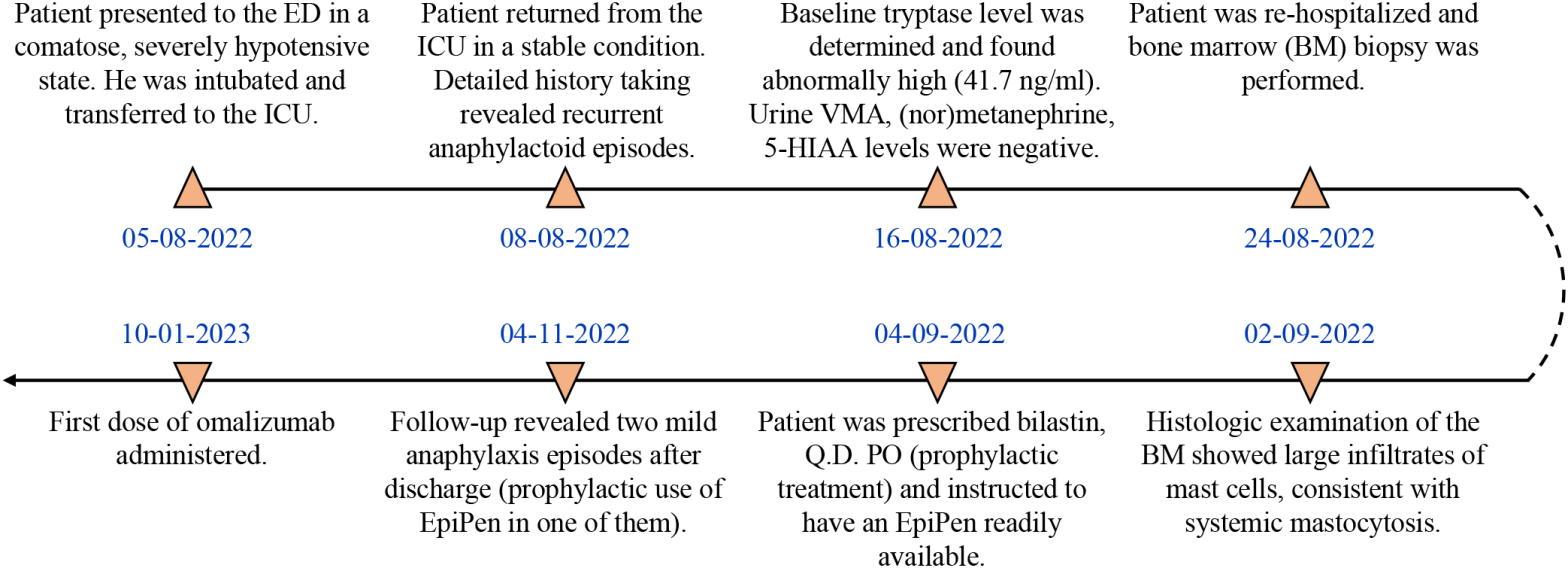
Timeline and case progress with relevant data.

A 45-year-old male patient (birthplace and residence: Athens, Greece) with a family and past medical history of arterial hypertension was admitted to the emergency department (ED) in a comatose state (Glascow Coma Scale; GCS: 3) after sudden loss of consciousness at work. The timeline of events and case progress with relevant data are presented in **Figure 1**.

**Figure 2:**
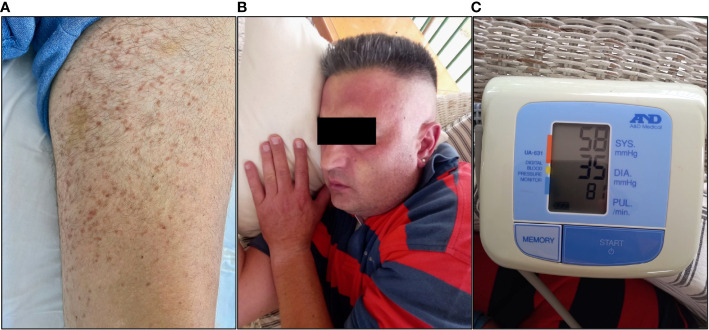
Photographs showing **(A)** the chronic urticarial rash, **(B)** facial flushing and **(C)** profound hypotension during an anaphylaxis episode in our patient (Courtesy of K.T.; written informed consent was obtained from the individual shown).

Upon arrival, the patient was hypotensive (BP: 70/40 mmHg) and was intubated shortly thereafter for airway protection. Assessment of cardiac function, including an ECG and echocardiogram, did not reveal any abnormalities, such as arrhythmia or severe hypokinesia that could explain the hypotension. Emergency contrast-enhanced CT scans of the brain (to exclude an intracranial hemorrhage), chest and abdomen (to exclude a dissecting aortic aneurysm) were also unremarkable. Clinical examination revealed mild hepatomegaly and multiple, monomorphic, reddish brown macules of the trunk and extremities (**Figure 2A**). There was no evidence of tongue biting or urinary incontinence, suggestive of a possible epileptic episode and postictal seizure state. Routine CBC and BMP were grossly unremarkable, except for a mildly elevated troponin level. Urine toxicology screen, consisting of benzodiazepines, barbiturates, opiates, amphetamines and cocaine was also negative. The patient was transferred to the ICU where he fully recovered within 48 hours. Upon recovery, the patient was able to recall several (~20) episodes of flushing (**Figure 2B**), tachycardia, lightheadedness and hypotension (**Figure 2C**) in the past, which he could link with emotional stress. Further inquiry about preceding events revealed a fairly consistent pattern of ingestion of certain foods (nuts, legumes, chocolate) or alcoholic beverages, together with emotional stress prior to the onset of the episodes. Intriguingly, these episodes continued even after the patient started establishing a link between them and food, gradually adjusting his diet by himself. Previous extensive work up in tertiary hospitals during the past 20 years, including negative food allergy testing, 24-hour Holter monitoring and electroencephalography, had failed to pinpoint to a specific cause for these episodes, other than psychological factors, thus recommending professional support.

**Figure 3:**
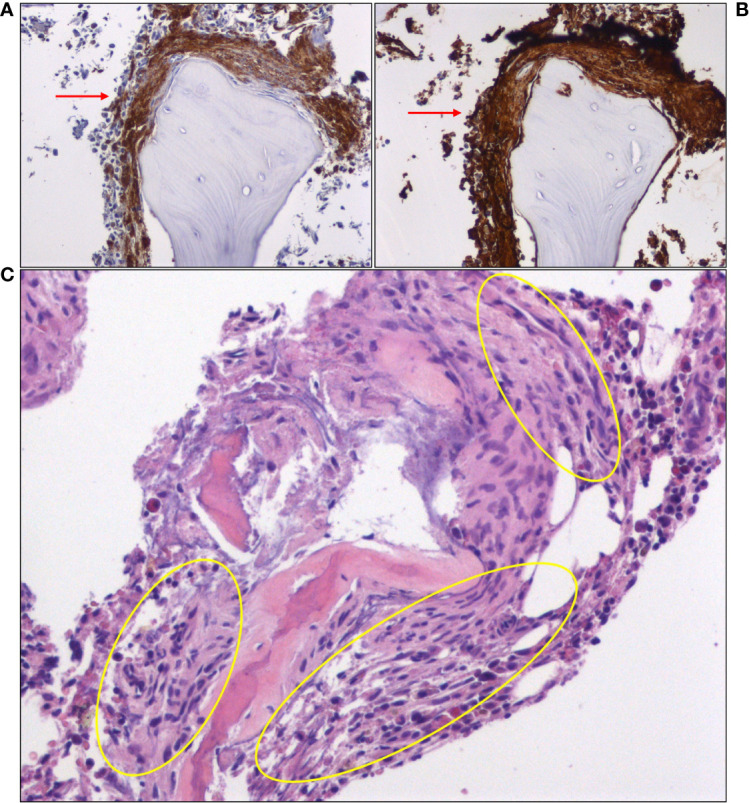
Immunohistochemical staining of bone marrow biopsy showing large clusters of mast cells (red arrows), expressing **(A)** CD25 and **(B)** mast cell tryptase. **(C)** A hematoxylin and eosin (H&E)-stained section of bone marrow core shows areas of paratrabecular fibrosis, in which mast cells can be identified (yellow oval circles).

Based on clinical presentation, imaging findings and previous workup, we assumed a non-cardiogenic, non-neurogenic cause for the recurrent anaphylactoid episodes, and turned our attention to endocrine disorders (pheochromocytoma, carcinoid syndrome) that can elicit such episodes, as well as SM. Testing for these rare diseases required external collaboration, as they were not routinely performed in our hospital. Urine VMA, (nor)metanephrine and 5-Hydroxyindoleacetic acid (5-HIAA) levels were normal, significantly reducing the probability of pheochromocytoma and carcinoid syndrome, respectively. Intriguingly, total serum tryptase level 10 days after the episode (baseline) was abnormally high (41.7 ng/ml; NR: < 11.4 ng/ml), fulfilling a minor SM criterion (**Table 1**). Subsequent bone marrow (BM) biopsy and histologic examination revealed an approximately 10% BM infiltration (multifocal dense infiltrates) by partially spindle-shaped CD117^+^/mast cell tryptase^+^/CD25^+^ MCs (**Figures 3A, B**) (major SM criterion; **Table 1**), establishing the diagnosis of SM. The observed mast cells had oval or elongated nuclei and were mostly located around bone marrow trabeculae (**Figure 3C**) and, to a lesser extent, blood vessels. The overall BM cellularity was normal (normal representation of the lymphoid lineage, mild elevation of the erythroid lineage and lower than normal myeloid lineage). Low grade paratrabecular fibrosis was evident in the stroma. Next generation sequencing on DNA extracted from neoplastic CD25^+^/CD2^+^ MCs following cell sorting did not reveal point mutations of the KIT (e.g. D816V mutation) and other genes (e.g. ASXL1, CBL, JAK2, RAS, RUNX1, SRSF2) that have been associated with SM, especially the advanced forms. The presence of typical chronic skin lesions (urticaria pigmentosa), along with the identification of only one B-finding (hepatomegaly without impairment of liver function) and no C-findings classified our SM case as ISM (favorable prognosis).

He was placed on a low histamine diet and started prophylactic treatment with a generally well-tolerated, non-sedating second-generation histamine H1-receptor antagonist (once daily). Since discharge, he has reported a couple of mild anaphylactic episodes, both in response to emotional stress. He pre-emptively used an EpiPen injection in one of them (as instructed to prevent recurrence of a shock condition). He also very recently started therapy with the anti-IgE monoclonal antibody omalizumab, which has shown promise in patients with ISM (5, 6).

## 3 Discussion

We describe a case of ISM with an unusually fulminant presentation leading to a comatose state and intubation. Our patient had remained undiagnosed for almost 20 years despite extensive previous neurological and cardiovascular workup, which was associated with a considerable financial burden.

ISM represents the most common form of SM, and is associated with anaphylactic reactions that can cause syncope in up to a third of all cases (7). However, very severe (grade 5) or fatal anaphylaxis (anaphylactic shock, GCS: <13) in ISM patients is rare. Review of the literature identified very few such cases, which occurred in response to either drug injection (iodinated contrast agents (8), anesthetics (9–11)) or insect stings (12–14). Unlike these reports, in our patient, severe anaphylactic reactions were precipitated by a combination of environmental (food) and emotional stimuli, also known risk factors for anaphylaxis in patients with mastocytosis (15). To the best of our knowledge, this is the first report of food- and emotional stress-induced anaphylactic shock in a patient with ISM. Diagnosis of ISM was based on a number of clinical and laboratory criteria, in accordance with the latest consensus guidelines. A limitation of our study is that we were unable to identify the highly prevalent KIT D816V mutation (or mutations in other SM-relevant genes), despite using sensitive molecular techniques, which suggests our patient may fall into the much less common KIT-negative category. Tryptase level could have been measured in the first few hours after the episode and compared with baseline tryptase in order to further strengthen our theory of persistent mast cell activation.

In conclusion, our case emphasizes that caution should be exercised by physicians when approaching conditions with systemic manifestations in order to exclude underlying organic causes, before attributing them solely to psychological factors. It further highlights the importance of detailed history taking, a largely neglected skill in modern medicine, in uncovering the recurrent nature of the episodes and identifying preceding events in diseases, such as SM. Careful physical examination of our patient also provided important clues (observation of the “typical” skin rash, absence of profound hepatosplenomegaly and lymphadenopathy). Lastly, in the case of SM, a high level of suspicion is required for its diagnosis, especially the indolent variant, due to its often-atypical presentation and slowly progressing clinical course.

Overall, our patient has met the developments in his health condition with enthusiasm, realizing he can finally consider an evidence-based approach to his long-standing symptoms. He has already experienced an improvement in his quality of life with the aforementioned interventions, while eagerly anticipating further benefit from omalizumab treatment.

## Data availability statement

The original contributions presented in the study are included in the article/supplementary material. Further inquiries can be directed to the corresponding author.

## Ethics statement

Written informed consent was obtained from the individual(s) for the publication of any potentially identifiable images or data included in this article.

## Author contributions

Article design and writing: AB, VT. Collection of clinical data/samples: IM, AB, DM, GiK, AL, VT. Immunohistochemistry procedures and pathology report: GeK. Supervision: AL, VT. All authors contributed to the article and approved the submitted version.
